# Will polygenic risk scores for cancer ever be clinically useful?

**DOI:** 10.1038/s41698-021-00176-1

**Published:** 2021-05-21

**Authors:** Amit Sud, Clare Turnbull, Richard Houlston

**Affiliations:** Division of Genetics and Epidemiology, The Institute of Cancer Research, London, UK

**Keywords:** Cancer genetics, Predictive markers, Cancer epidemiology, Cancer screening, Risk factors

Genome-wide association studies (GWAS) have identified associations between common genetic variants, single nucleotide polymorphisms (SNPs) and the risk of developing different cancers^1–3^. Proponents argue that polygenic risk score (PRS) testing, based on panels of risk SNPs, will revolutionize the prevention and early detection of cancer through individualised risk management strategies and streamlining of the current ‘one-size-fits all’ population screening programs^4^. Such a model is highly seductive for the rationalisation of healthcare provision. UK government enthusiasm for PRSs is well demonstrated within the recent Genome UK report and 2020 update to the Life Sciences Strategy^5^. Indeed, reflecting governmental endorsement of predictive genomics, the UK government’s Secretary of State for Health and Social Care, Matthew Hancock, rather questionably enthused that his recent PRS-derived lifetime prostate cancer risk estimate of 15% (compared to a prior of 13%) “may have saved his life”^6,7^. To establish the requisite governance and data infrastructure for population-level genomic profiling, national projects such as the 100,000 Genomes Project (>70,000 NHS patients) and the Accelerating Detection of Disease programme (up to 5 million volunteers) were initiated^8,9^. Following initial discontinuation by the U.S. Food and Drug Administration of the 23andMe PRS service^10,11^, there has been a resurgence within the direct-to-consumer genomics market of PRS predictions for many diseases. While the value of additional biomarkers to improve the targeting of measures for cancer prevention and early detection is indisputable, for PRS to be clinically useful, two assertions must be proven correct. The first assertion is that PRSs provides sufficient risk discrimination. The second is that this risk discrimination is meaningful in the context of absolute risk of that cancer and applicable in the context of respective tools available for prevention and early detection.

The risk discrimination for a given cancer afforded by PRS can be visualised most simply via PRS frequency distributions of those with the disease compared to those without the disease. (Fig. 1)^12,13^. Two commonly presented measures of discrimination derived from these distributions are: (i) comparison of the RR for those at the top and bottom tails of the PRS distribution (ii) comparison for specified PRS cut-offs of the proportion of affected individuals with ‘positive’ PRS (‘detection rate’) versus the proportion of unaffected individuals falling within the same PRS score range (the ‘false-positive rate’ (1–specificity)) (Supplementary Fig. 1). For the common cancers, PRS for prostate currently leads on discriminatory performance, with RR of 14.54 between the top 5% and bottom 5% of men (Fig. 1)^14^. This translates to a false-positive rate of 5% for a detection rate of 16%. Or, to achieve a detection rate of 50%, tolerance of a false-positive rate of 25%.

**Fig. 1:**
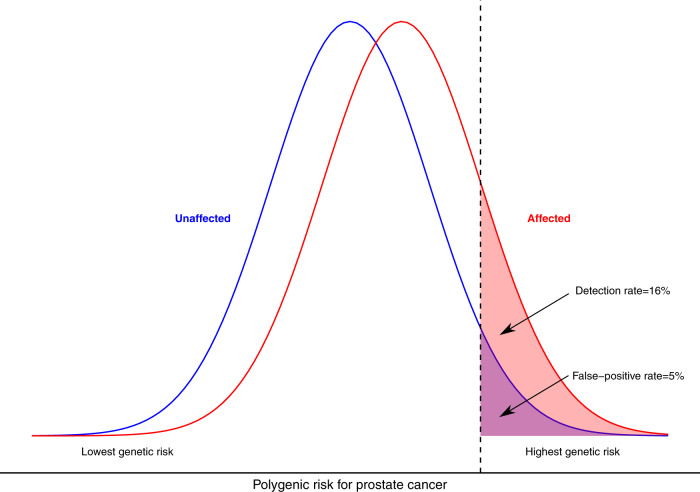
Overlapping relative frequency distributions of polygenic risk score in prostate cancer cases and unaffected individuals. The detection rate for a false-positive rate of 5% is 16%.

Quantitation of PRS performance tends to focus on the tails of the distribution, distracting from the fact that for 90% of individuals their PRS lies relatively close (<2 SD) to the mean. Up- or down-modification by these PRSs against baseline cancer risk results in minimal absolute difference in risk. Acknowledgement that the PRS for a chosen disease provides useful information for only a few percent of people is often countered by the argument that PRSs can be generated for dozens of cancer types, such that each individual is likely to be in an ‘extreme’ tail of PRS for at least one cancer type. However, in practice, clinical decision-making is driven by absolute risk, rather than relative risk per se. Hence, for all but the most common cancers, PRS reveals absolute increase in cancer risk against baseline that is miniscule even at the extreme upper tail of PRS. For example, for those in the respective top 5% of PRS, lifetime risk for breast cancer is elevated from 11.8 to 19.0% (1.6-fold), lifetime risk of prostate cancer is elevated from 12.7 to 22.2% (1.75-fold) and lifetime risk of colorectal cancer is elevated from 4.6 to 6.9% (1.5-fold). For less common cancers, the absolute risk increase is much lower: for women in the respective top 5% of ovarian cancer PRS lifetime risk is elevated 1.3-fold (from 1.6 to 2.1%)^14,15^.

A presentation popular for capturing the discriminatory attributes of PRS is the Receiver Operating Characteristic (ROC) curve, with the probability that a randomly selected case having a higher PRS than a randomly selected control being quantified by the area under the ROC curve (AUC). An AUC of 0.5 reflects a testing tool with no discrimination. While the threshold for clinical discrimination clearly depends on the benefit-risk profile of the proposed intervention, broadly speaking AUC values of 0.7–0.8 are considered as acceptable and >0.8 as affording good discrimination^16^. As an example, digital mammography for breast cancer screening has an AUC of 0.78^17^. PRSs constructed from cancer SNP sets derived from GWAS yield only AUCs ranging from 0.53 (renal cancer) to 0.67 (prostate cancer) (Fig. 2).

**Fig. 2:**
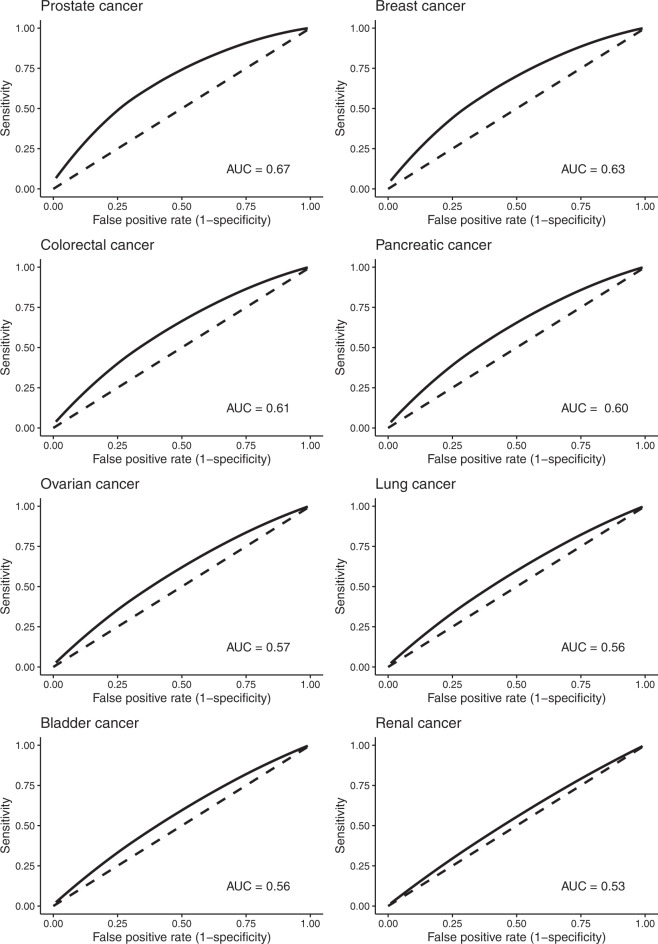
Receiver operator plots of polygenic risk scores for eight common cancers. AUC area under the curve. The AUC provides an estimate of the probability a randomly selected subject with the condition has a test result indicating greater than that of a randomly chosen individual without the cancer. The solid line represents a receiver operator curve based on polygenic risk score from known risk SNPs based on reference^13^. An AUC of 0.5 (dashed line) indicates that the classifier does not provide any useful information in discriminating cases from controls.

The GWAS so far performed have only identified SNPs contributing 10–30% of common variant heritability for their respective cancers. It is widely asserted that discriminatory value of PRS will improve dramatically ‘in the future’, once additional rafts of new disease-associated SNPs are delivered. Imposing a threshold of 5 × 10^−8^ for declaring a SNP association guards against type 1 error but inevitably has limited the number of SNPs included in PRS testing panels. Methodologies such as LDpred apply a Bayesian genome-wide genetic risk prediction to incorporate SNP-correlations “tagged” by current arrays irrespective of association *P*-value^18^. However, to the extent that such methods have been explored, the discriminatory power for these ‘expanded’ PRSs is at best only very modestly improved^19,20^. The (likely) final generation of GWASs for common cancers such as breast and colorectal cancers is currently underway. Despite aggregating the’world-wide’ resource of available samples, power remains prohibitive and these experiments are unlikely to harvest more than 80% of the heritable risk for these cancers^3^. Even disregarding this and calculating PRS based on the full disease heritability, i.e. the hypothetical situation of identifying the full complement of risk SNPs, the AUCs for the common cancers remain disappointingly modest (0.64–0.73)^3^.

Another argument pertains to the boost in predictive value of PRS when applied in combination with non-genetic risk factors. Family history can be an important risk factor for cancer but is correlated with PRS^21–23^; some authors erroneously inflate their AUC by combining the two as if orthogonal^24,25^. For most cancer types, the AUC for non-genetic factors is modest^26,27^. When modelled, the well-established modifiable risk factors have additive effects with SNP associations and therefore only modestly improve the AUC^27,28^. Breast cancer has the best characterised set of non-genetic risk factors: the AUC for these risk factors is 0.637 while the AUC of the current PRS is 0.631; and in combination the AUC is 0.683^29^. Again, while aetiological epidemiological research continues to be a dynamic field, it would seem naïve to predict imminent discovery of robustly associated new non-genetic risk factors of sizeable effect.

There are many who acknowledge PRS to be a weak predictor of individual cancer risk but who suggest that it could still be useful to individualise population screening programmes, for example by excluding ‘low risk’ individuals from screening^30,31^. From modelled adaptation of the UK breast cancer screening programme such that screening of women age 35–79 would be offered on the basis of PRS rather than age alone, it has been predicted that screening could be reduced by 24% at the cost of reduction in screen-detectable cases of 14%^32^. Although this indicates potential for more efficient targeting of screening, there is concern that: (i) genomic profiling will do little to improve and could even reduce uptake of existing cancer screening programmes, which for breast cancer in the UK is currently only 69%^33^, and (ii) the incidence of breast cancers in those from whom breast screening has been ‘withheld’ (or in fact ‘withdrawn’) would be perceived by the public as too high. Another proposal is that PRSs could be combined as a Bayesian Prior to a population screening test, such that the more expensive and/or invasive confirmatory test is only triggered when a positive screening test (PSA, FIT) arises in individuals with high PRS. In practice, where the screening test itself has good performance characteristics, for example Faecal Immunochemical Test (FIT) for detection of colorectal cancer, the negative predictive value of PRSs will be insufficient to exclude disease in those with a positive screening test. Conversely, where the performance of the screening test is poorer (e.g. prostate specific antigen (PSA) for prostate cancer), the limited positive predictive value of the PRS adds little, resulting in a large group of PRS-false-positives receiving an invasive, confirmatory investigation. Hence, where screening is effective, inexpensive and safe, the limited additional boost in detection added by PRS stratification is arguably likely to be outweighed by the cost, complexity of delivery of population risk-profiling with the potential for reduced participation. Well-designed trials are therefore essential to avoid inadvertent disruption of existing screening programmes with documented evidence of benefit^34^. Where screening is not effective, inexpensive, or safe, the addition of a PRS is unlikely to make it so.

PRS testing has been suggested as a method of identifying individuals who may benefit from cancer chemopreventative agents^35^. While an attractive proposition, few agents are currently licenced for this purpose and clinical studies of new agents for chemoprevention are challenging. The risk-benefit profile of an agent dictates the level of disease risk at which administration is justified; thus the value of PRS stratification in determining administration would be very much contingent on the performance of PRS in discriminating those at sufficiently elevated risk. Administration of chemopreventative agents based on PRS-stratified groups has not yet been trialled for any agents and would require careful consideration^36,37^. While communicating DNA-based disease risk assessments may have a role in promoting risk-reducing behaviour^38^, evidence for this is currently lacking and there is a potential to introduce harm, particularly in the absence of counselling and clinical utility^39^. Furthermore, if PRS is to be used, it has to be universally applicable to all in the population regardless of ancestry to ensure equity in provision of healthcare resource. Presently the majority of PRSs are based on studies of European ancestry^40^ and their performance is poor in non-European populations^41^. This consideration is frequently offered as a minor technical ‘footnote’ to the value proposition of PRSs: given the limited predictive capabilities of existing PRS generated from decades of massive studies of available samples, it is unclear how this deficit will actually be addressed in the foreseeable future.

The clinical utility of identification of high-impact mutations in genes such as *BRCA1* and *MLH1* is not under dispute: such mutations provide effective risk discrimination and there are established clinical pathways for those in whom mutations are identified. However, the notion that PRSs will offer equivalent utility population-wide by providing informative risk stratification across multiple diseases is misleading. Raising unrealistic expectations and implementing programmes without careful evaluation risks compromising the application of PRSs for specific niches, and indeed, of genomic medicine as a whole.

## Supplementary information

Supplementary Information

## Data Availability

No datasets were generated or analysed during the current study.
