# Supplementary material for: Will polygenic risk scores for cancer ever be clinically useful?
Source: NPJ Precis Oncol. 2021 May 21;5:40. doi: 10.1038/s41698-021-00176-1 (PMC8139954; doi:10.1038/s41698-021-00176-1)
Supplement: Supplementary file 1 — Supplementary Information [file 41698_2021_176_MOESM1_ESM.pdf]

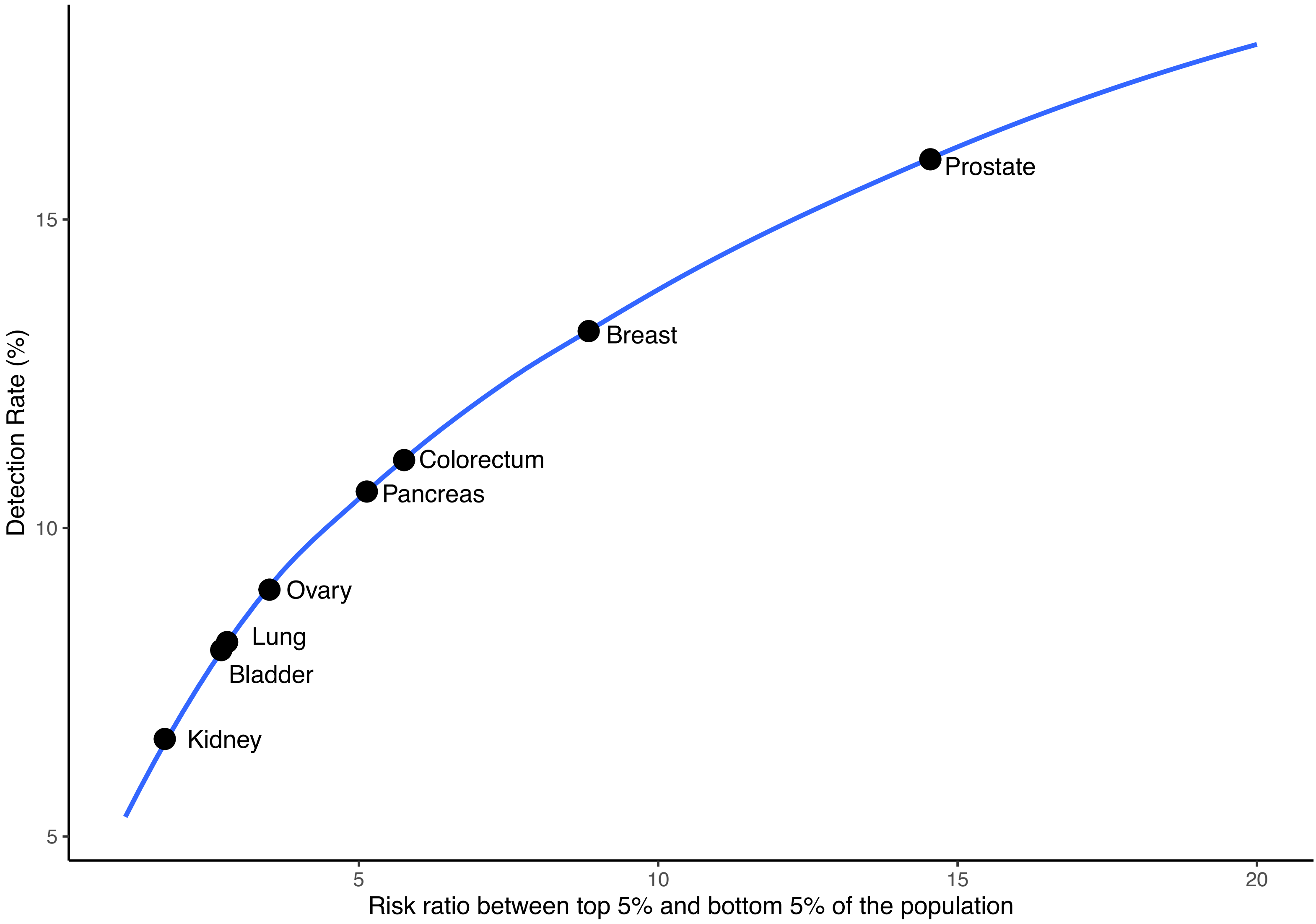

Supplementary Figure 1. Probability of cancer (detection rate) according to PRS defined relative risk for each common cancer. Polygenic risk score - >95% centile compared with <5th centile. Analysis conducted using the risk screening converter tool[13].
